# Trajectories of gray and white matter volume in children at elevated risk for schizophrenia

**DOI:** 10.1017/S1092852926101047

**Published:** 2026-06-09

**Authors:** Shuqing Si, Matthew Kempton, Emily Hedges, Mathilde Antoniades, Ruth E. Roberts, Alexis E. Cullen, Kristin R. Laurens

**Affiliations:** 1 Department of Psychosis Studies, King’s College London, UK; 2 Center for AI and Data Science for Integrated Diagnostics, and Center for Biomedical Image Computing and Analytics, University of Pennsylvania, USA; 3 Education & Training Division, Anna Freud Centre, UK; 4 Research Department of Clinical, Educational & Health Psychology, University College London, UK; 5 National Institute for Health Research (NIHR), Mental Health Biomedical Research Centre at South London and Maudsley NHS Foundation Trust and King’s College London, UK; 6 Division of Insurance Medicine, Department of Clinical Neuroscience, Karolinska Institutet, Sweden; 7 School of Psychology and Counselling, Queensland University of Technology (QUT), Australia; 8 Social, Genetic & Developmental Psychiatry Centre, King’s College London, UK

**Keywords:** high risk, psychosis, structural MRI, community screening, early detection, longitudinal study

## Abstract

**Objective:**

Longitudinal investigation of brain alterations in children putatively at risk of developing schizophrenia may identify early markers of pathophysiology associated with vulnerability for the disorder.

**Methods:**

Children aged 9–12 years with multiple antecedents of schizophrenia (psychotic-like experiences; speech and/or motor delays; and social, emotional, and/or behavioral difficulties; ASz, *n* = 31), with a family history of schizophrenia/schizoaffective disorder (FHx, *n* = 21), and typically developing children (TD, *n* = 35) were recruited through community screening using questionnaires. T1-weighted structural MRI scans were obtained at baseline and after approximately 2 and 4 years of follow-up, and processed using the FreeSurfer longitudinal pipeline. Across these 3 assessment waves, total gray matter (GM) and white matter (WM) volume, and regional GM volumes in 12 regions of interest, were compared among the groups using linear mixed models.

**Results:**

Children were aged 11.21 (±1.61) years at baseline and the average lapse-of-time between each assessment was 1.76 (±0.36) years. The ASz group exhibited consistently higher total WM volume (*b* = 29.45) compared to the TD group, with no statistically significant time-related volumetric WM or GM differences in any region. The FHx group also demonstrated higher total WM volume (*b* = 36.49), and time-related total and regional GM volume changes were observed; however, these effects were attenuated after correction.

**Conclusion:**

Global and regionally specific brain abnormalities distinguished children with different risk profiles for schizophrenia relative to their typically developing peers. Follow-up is required to determine how these changes may relate to later schizophrenia.

## Introduction

Brain abnormalities among individuals with schizophrenia and related psychotic disorders have been consistently reported across many studies.^1^^–^^4^ These abnormalities include reductions in whole brain volume relative to healthy individuals, and regional volume reductions affecting the frontal and temporal lobes, particularly in the superior temporal gyri, insulae, and hippocampus. Such abnormalities are likewise present among individuals experiencing their first episode of psychosis,^5^ prompting investigations of whether these abnormalities may precede illness onset.

Over the past 3 decades, efforts have focused on identifying individuals at increased risk of psychosis who may benefit from early intervention, thereby minimizing the potential confounding effects on brain structure from antipsychotic medication.^6^ The 2 most commonly used approaches are based on family history of psychotic disorders (FHR) or the presence of attenuated psychotic symptoms (clinical high risk [CHR] for psychosis).^7^ Studies adopting the FHR approach have yielded variable transition rates (15–40%),^8^ while meta-analyses in CHR individuals report ~22% transition over 2 years.^9^^–^^11^ Both groups show subtle brain structural alterations,^12^^,^^13^ though in different regions. A meta-analysis of participants aged 12 years or older (mean age > 20)^14^ reported decreased GM in insula and caudate in CHR individuals, while FHR individuals showed reductions in anterior cingulate, inferior frontal, and temporal cortices. A small number of studies have reported white matter (WM) volume differences; a review^15^ highlighted that 3 studies^16^^–^^18^ reported larger baseline WM volume, and decreases in volume or smaller increases over time, in at-risk individuals (both CHR and FHR) compared to healthy controls.

Studies of FHR and CHR individuals have important limitations. Although heritability contributes substantially, many individuals with psychosis lack a family history,^19^ limiting the specificity of FHR-based identification. CHR individuals are, by definition, help seeking with functional impairments^20^; consequently, this method may miss at-risk individuals who do not access health/support services. Moreover, individuals at CHR have comorbidities, such as depression or anxiety disorder, that are themselves associated with brain structure alterations.^21^^,^^22^

A more informative approach may be to study at-risk youth at an earlier stage – prior to CHR status – to capture neurodevelopmental deviations before help seeking or clinical impairment emerges.^23^ Here, we present longitudinal MRI data from the London Child Health and Development Study (CHADS) which followed putatively at-risk individuals recruited from the community during the premorbid phase, before the advent of the significant functional impairment.^24^

CHADS adopted 2 main approaches to identifying at-risk children aged 9–12 years.^25^ The first approach utilized novel community screening methods to identify children with multiple antecedents of schizophrenia (ASz). Psychotic-like experiences (PLEs) in childhood indicate elevated risk for later psychopathology,^26^^,^^27^ and were combined with 2 additional risk factors, namely speech and/or motor development delays, and social, emotional, and/or behavioral difficulties, to form a triad of well-replicated antecedents of schizophrenia. A previous cross-sectional imaging study using data from this cohort reported that ASz children aged 9–12 years showed decreased GM volume in the right middle temporal gyrus (MTG) and increased GM volume in the left superior-middle temporal gyrus relative to their typically developing (TD) peers who had no family history or antecedents of schizophrenia, as well as increased WM clusters in parietal, occipital, and temporal regions.^28^

The second risk identification approach adopted in the CHADS study, based on family history (FHx), involved identifying child relatives (first or second degree) of patients with schizophrenia. Although brain structural abnormalities have not been examined previously in this group, other longitudinal analyses in the CHADS cohort revealed that ASz and FHx groups each showed different developmental trajectories of brain function^29^ and cognitive function,^30^ compared to the TD group.

To explore trajectories of brain structure in at-risk children, this study used longitudinal MRI data obtained at 3 assessments completed during childhood and adolescence. Building on existing research, we hypothesized that at-risk children in both the ASz and FHx groups would present with altered global GM and WM volume as well as regional GM volumes, and different trajectories of brain development, compared to the TD group.

## Methods

### Participants

- The longitudinal CHADS project, including details of the various assessments completed at each wave (including imaging), has been described in detail elsewhere.^25^^,^^31^ Children were first enrolled into the study at the age of 9–12 years (referred to as baseline assessment, BL), and follow-up assessments were performed at approximately 2 years after baseline (referred to as follow-up 1, FU1; age ~ 11–14 years) and 4 years after baseline (referred to as follow-up 2, FU2; age ~ 13–16 years).
- Children with a heightened risk of developing schizophrenia presented with either a triad of antecedents of schizophrenia (ASz) or a family history of schizophrenia or schizoaffective disorder (FHx). The triad of antecedents was defined as: (1) at least one child-reported “certainly true” response to any item on the 9-item Psychotic-Like Experiences Questionnaire for Children (PLEQ-C^32^^,^^33^); (2) child-reported emotional symptoms or caregiver-reported conduct problems, hyperactivity inattention, or peer relationship problems in the clinical range (approximately top 10th percentile on the United Kingdom norms) assessed using the Strengths and Difficulties Questionnaire^34^; and (3) a caregiver-reported delay (in attaining developmental milestones within the first 3 years of life) and/or abnormality (based on caregiver or professional expressions of concern) in speech and/or motor development^24^ (see Supplementary Material for additional detail regarding antecedent assessment). Participants who met all 3 antecedent criteria were recruited to the ASz group. Children who had relatives with schizophrenia or schizoaffective disorder were identified from caregiver questionnaires or via review of patients’ medical records within the South London and Maudsley National Health Service (NHS) Foundation Trust, with this family history confirmed using the Family Interview for Genetic Studies (FIGS).^25^ Participants with at least one affected first- or second-degree relative were recruited into the FHx group. Children who did not present with any of the 3 antecedents and who had no family history of schizophrenia or related severe mental illness in any first-, second-, or third-degree relative were defined as typically developing (TD). Specified exclusion criteria for study participants included: (i) insufficient English language ability (child or caregiver) to complete assessments; (ii) a neurological condition affecting milestone attainment or current functioning (eg, epilepsy, cerebral palsy); (iii) a diagnosis of autism, Asperger’s disorder, or learning disability (IQ < 70); and (iv) a prior psychotic episode or use of antipsychotic medication. All children were also screened via the FIGS^25^ (though not excluded) for family history of bipolar disorder, personality, substance use, and major depressive disorders (see Supplementary Material for further detail). In this study, children who fulfilled criteria for both ASz and FHx groups were preferentially assigned to the ASz group in primary analyses. This maximized statistical power to characterize pathological trajectories among individuals who were identified via the novel community screening approach as demonstrating early phenotypic expression of risk. (Sensitivity analyses were conducted to observe any change to findings after reassigning these individuals who met the criteria of both groups from the ASz to the FHx group.)
- Written assent and consent were obtained from the children and caregivers, respectively, at each wave of assessment. Ethical approval of the study was obtained from the Joint South London and Maudsley and Institute of Psychiatry Research Ethics Committee.

### MRI acquisition and image processing

Longitudinal structural MRI scans were acquired on a 3T GE Medical Systems MRI scanner (GE Healthcare) using a 3-dimensional Spoiled Gradient Recalled Echo sequence (repetition time [TR] = 6.0 ms, echo time [TE] = 2.8 ms, flip angle = 20°, 256 × 256 matrix size, yielding 196 slices covering the whole brain with a thickness of 1.1 mm). The structural images were processed using FreeSurfer v7.1.0 (http://freesurfer.net) using the “recon-all” longitudinal pipeline with the default set of parameters. Of the 273 scans available from 115 participants, 5 (1.8%) were excluded during an initial manual quality check due to movement artefacts. Quality assessment was performed using the ENIGMA Cortical QC Protocol (http://enigma.usc.edu), and a further 32 (11.7%) scans were excluded due to poor segmentation, leaving a total of 236 MRI images from 88 participants.

We utilized total GM and WM to capture global structural brain changes. For region-specific analyses, we selected regions of interest (ROIs) based on consistent evidence from large-scale studies of schizophrenia and first-episode psychosis. These included the insula, fusiform gyrus, anterior cingulate cortex (ACC), and hippocampus, which have shown robust structural alterations.^2^^,^^4^^,^^35^^–^^38^ Total WM volume was calculated by summing cerebral WM and cerebellum WM from the ROI output of FreeSurfer. In addition, the middle and superior temporal gyri (MTG, STG) were included based on prior findings from the CHADS cohort demonstrating altered volumes in these regions at baseline.^28^

### Statistical analysis

Statistical analyses were performed in Stata version 18 (StataCorp., 2021). The BL characteristics of ASz, FHx, and TD groups were compared using chi-squared or Kruskal–Wallis tests, as appropriate.

To investigate changes in brain volume between the ages of 9 and 16 years, mixed effects models (using the “mixed” command) were fitted for global volume and each of the 12 brain regions individually (ie, with each regional brain volume as the dependent variable). These models optimally accommodate missing data and small sample sizes.^39^ The main predictor in all statistical models was group status at recruitment (TD vs. ASz vs. FHx). Additional covariates included assessment wave (BL vs. FU1 vs. FU2, entered as categorical variables) to represent time within the study and an interaction between group and time (group × assessment wave). Waves were used, instead of age, as the time variable to simplify the model given the limited sample size, and to better capture average within-subject changes across assessment points. Age at BL (date of experiment minus date of birth) was included as a continuous covariate to account for variations in participant age when recruited to the project, and a variable coding days lapsing from BL was included to account for participant differences in timing between each follow-up session. Sex (female, male) and total intracranial volume (ICV) were additionally covaried in all analyses. Unfortunately, the small sample of participants available for analysis precluded consideration of other covariates that might relate to brain volume alterations among at-risk children (eg, measures of cognitive function, adverse experiences).

Individuals were fitted in the model as random effects to account for repeated measures from the same individual. All random intercept models were fitted using maximum likelihood estimation. The interaction term between group and wave was first included in all models and subsequently eliminated if it was not a statistically significant predictor (*p* < 0.05), and a simpler model with only main effects was selected.^40^ Unstandardized b coefficients and 95% confidence intervals (CI) are reported for each variable. To adjust for multiple comparisons, we used false discovery rate (FDR) correction. All 84 *p* values from main fixed effects of mixed effects models were included in the correction. Marginal and conditional *R*^2^ are reported for each model to show the proportion of variance explained by fixed or fixed and random effect, respectively. The multicollinearity between all variables included in the model was checked using the “collin” command. The “margins” command was used to calculate predictions from each model and generate margins plots for brain regions where a significant effect of group status was observed. In analyses that showed a significant main effect of risk group status, pairwise comparisons were then performed using “margins pwcompare,” with default options to compare the brain volumes between groups at each time point. Furthermore, we performed sensitivity analyses in which individuals who met the criteria for both ASz and FHx groups (*n* = 5) were reassigned from the ASz to the FHx group.

## Results

### Demographic characteristics

A total of 236 MRI images from 88 participants were analyzed, comprising 35 TD, 32 ASz, and 21 FHx individuals with at least one available MRI scan (Table 1). The mean (±SD) age at the baseline scan across the total sample was 11.21 years (±1.61) and the lapse of time between each assessment of 1.76 years (±0.36). Among all participants, 18 had a single scan, 32 had 2, and 38 had all 3 MRI scans available. There were no significant differences in sex or age among the 3 groups at each assessment (Table 1).

**Table 1.** Demographic information of TD, ASz, and FHx groups Table 1. long description.

	TD (*n* = 35)	ASz (*n* = 32)	FHx (*n* = 21)	*p*
Number of participants with an MRI scan available, by assessment wave				
BL	21 (60%)	24 (75%)	12 (57%)	
FU1 (~2 years)	32 (91%)	23 (72%)	14 (67%)	
FU2 (~4 years)	30 (86%)	26 (81%)	14 (67%)	
Sex, female (%)	20 (57%)	10 (31%)	12 (57%)	0.07
Age (years), mean ± SD				
BL	11.03 ± 0.14	11.18 ± 0.24	11.56 ± 0.27	0.15
FU1 (~2 years)	12.99 ± 0.17	12.81 ± 0.25	13.52 ± 0.20	0.07
FU2 (~4 years)	14.78 ± 0.19	14.50 ± 0.22	15.03 ± 0.22	0.23

Abbreviation: TD, typically developing group; ASz, antecedent group; FHx, family history group; BL, baseline; FU1, follow-up 1; FU2, follow-up 2.

Results for the main effects of risk group (ASz vs. TD; FHx vs. TD), assessment wave (FU1 vs. BL; FU2 vs. BL), and their interactions are shown in Table 2 for total GM/WM and for each ROI. The interaction between risk group and wave was not a significant predictor for the left hippocampus, left and right fusiform gyri, left and right ACC, and left and right STG, and was consequently dropped from the final model for these regions.

**Table 2.** Parameter estimates of the effect of risk group on gray and white matter volumes over the 3 waves of assessment Table 2. long description.

		Estimated between-group differences over 4-years (group effect)				Estimated change over assessment wave (wave effect)				Estimated between-group differences in change between respective assessment waves (group × wave)							
		ASz vs. TD		FHx vs. TD		FU1 vs. BL		FU2 vs. BL		ASz × FU1		ASz × FU2		FHx × FU1		FHx × FU2	
Regions of interest	*R*^2^	*b*	95% CI	*b*	95% CI	*b*	95% CI	*b*	95% CI	*b*	95% CI	*b*	95% CI	*b*	95% CI	*b*	95% CI
Total GM	Conditional *R*^2^ = 0.89 Marginal *R*^2^ = 0.72	8.388	−12.959 to 29.735	7.538	−17.803 to 32.878	11.558	−13.044 to 36.160	5.462	–36.311 to 46.236	1.734	−18.415 to 21.883	3.836	−16.446 to 24.134	−26.200*	−51.388 to −1.010	−5.031	−30.303 to 20.241
Total WM	Conditional *R*^2^ = 0.99 Marginal *R*^2^ = 0.80	29.452**	14.913 to 43.992	36.494**	20.052 to 52.935	15.262**	6.397 to 24.126	23.809**	8.578 to 39.041	−0.708	−6.807 to 5.390	−2.873	−9.034 to 3.289	−11.813**	−19.656 to −3.969	−14.549**	−22.347 to −6.752
Left hippocampus	Conditional *R*^2^ = 0.93 Marginal *R*^2^ = 0.45	−0.052	−0.225 to 0.122	−0.058	−0.252 to 0.136	−0.063	−0.197 to 0.071	−0.167	−0.405 to 0.072	NA	NA	NA	NA	NA		NA	
Right hippocampus	Conditional *R*^2^ = 0.94 Marginal *R*^2^ = 0.43	−0.037	−0.246 to 0.172	0.064	−0.174 to 0.303	0.082	−0.077 to 0.241	0.064	−0.207 to 0.336	−0.023	−0.136 to 0.089	0.004	−0.109 to 0.118	−0.171*	−0.314 to −0.027	−0.039	−0.182 to 0.104
Left fusiform	Conditional *R*^2^ = 0.94 Marginal *R*^2^ = 0.36	0.116	−0.501 to 0.734	−0.106	−0.795 to 0.583	0.208	−0.196 to 0.612	0.098	−0.624 to 0.820	NA	NA	NA	NA	NA		NA	
Right fusiform	Conditional *R*^2^ = 0.91 Marginal *R*^2^ = 0.34	0.310	−0.235 to 0.854	0.268	−0.340 to 0.877	0.044	−0.385 to 0.474	−0.291	−1.055 to 0.474	NA	NA	NA	NA	NA		NA	
Left insula	Conditional *R*^2^ = 0.94 Marginal *R*^2^ = 0.54	0.078	−0.234 to 0.391	0.190	−0.168 to 0.549	0.052	−0.205 to 0.308	−0.023	−0.460 to 0.415	0.04	−0.144 to 0.224	0.138	−0.048 to 0.323	0.233	−0.002 to 0.468	0.246*	0.011 to 0.480
Right insula	Conditional *R*^2^ = 0.92 Marginal *R*^2^ = 0.53	−0.048	−0.380 to 0.285	0.247	−0.137 to 0.631	−0.241	−0.543 to 0.061	−0.434	−0.947 to 0.079	0.099	−0.123 to 0.322	0.112	−0.113 to 0.336	0.364*	0.081 to 0.647	0.202	−0.080 to 0.484
Left ACC	Conditional *R*^2^ = 0.97 Marginal *R*^2^ = 0.48	0.071	−0.364 to 0.506	0.206	−0.279 to 0.691	0.273*	0.047 to 0.498	0.408*	0.004 to 0.812	NA	NA	NA	NA	NA		NA	
Right ACC	Conditional *R*^2^ = 0.97 Marginal *R*^2^ = 0.20	0.025	−0.357 to 0.407	0.078	−0.348 to 0.503	0.176*	0.007 to 0.345	0.193	−0.109 to 0.496	NA	NA	NA	NA	NA		NA	
Left MTG	Conditional *R*^2^ = 0.84 Marginal *R*^2^ = 0.44	0.163	−0.732 to 1.057	0.178	−0.873 to 1.229	0.403	−0.555 to 1.362	−0.064	−1.670 to 1.542	0.252	−0.498 to 1.002	0.274	−0.482 to 1.030	−1.062*	−2.008 to −0.117	−0.442	−1.388 to 0.504
Right MTG	Conditional *R*^2^ = 0.84 Marginal *R*^2^ = 0.37	0.502	−0.533 to 1.537	0.327	−0.882 to 1.536	0.669	−0.391 to 1.729	−0.168	−1.953 to 1.617	−0.102	−0.914 to 0.710	0.035	−0.784 to 0.855	−1.124*	−2.152 to −0.097	−0.280	−1.307 to 0.747
Left STG	Conditional *R*^2^ = 0.93 Marginal *R*^2^ = 0.39	0.479	−0.282 to 1.240	0.462	−0.389 to 1.312	0.374	−0.172 to 0.920	0.375	−0.599 to 1.349	NA	NA	NA	NA	NA		NA	
Right STG	Conditional *R*^2^ = 0.94 Marginal *R*^2^ = 0.39	0.230	−0.486 to 0.946	0.764	−0.036 to 1.563	0.094	−0.414 to 0.601	−0.074	−0.979 to 0.832	NA	NA	NA	NA	NA		NA	

*p < 0.05; **FDR corrected *p* < 0.05; significant effects in boldface; Abbreviation: TD, typically developing group; ASz, antecedent group; FHx, family history group; NA, interaction between group and time was not significant and was dropped from the model; GM, total gray matter volume; WM, total white matter volume; ACC, anterior cingulate; MTG, middle temporal gyrus; STG, superior temporal gyrus; BL, baseline; FU1, follow-up 1; FU2, follow-up 2.

### Main effect of group and assessment wave

**Figure 1.**
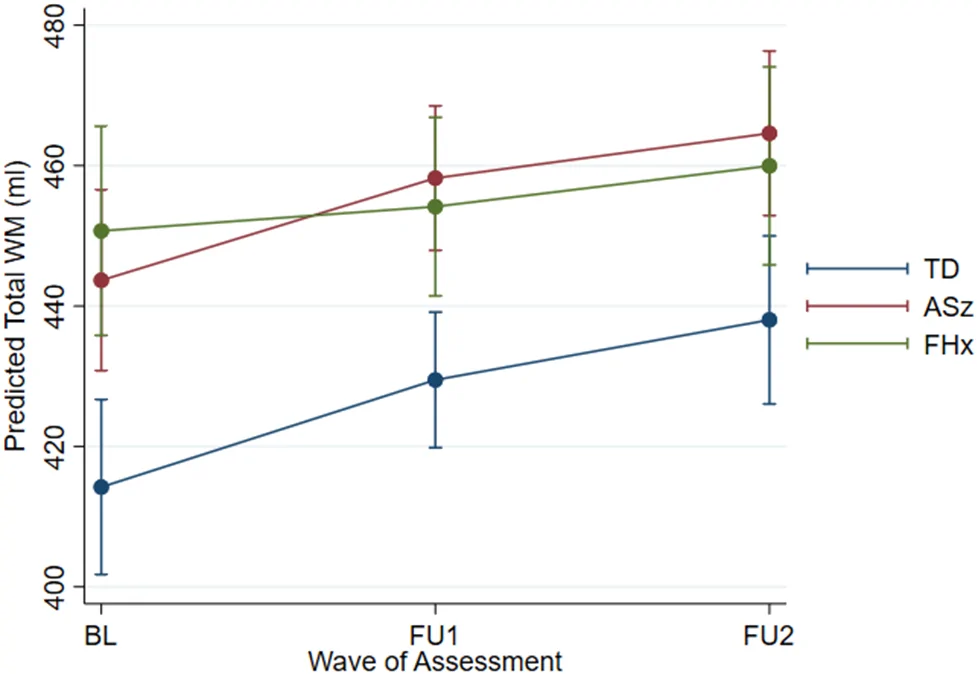
Margin plots of predicted total white matter (WM) volume with 95% confidence intervals for the 3 groups showing changes over 3 waves of assessment, adjusted for age at baseline, days lapsing from baseline, sex, and intracranial volume. TD: typical development group; ASz: antecedent group; FHx: family history group; BL: baseline; FU1: follow-up 1; FU2: follow-up 2. Figure 1. long description.

**Figure 2.**
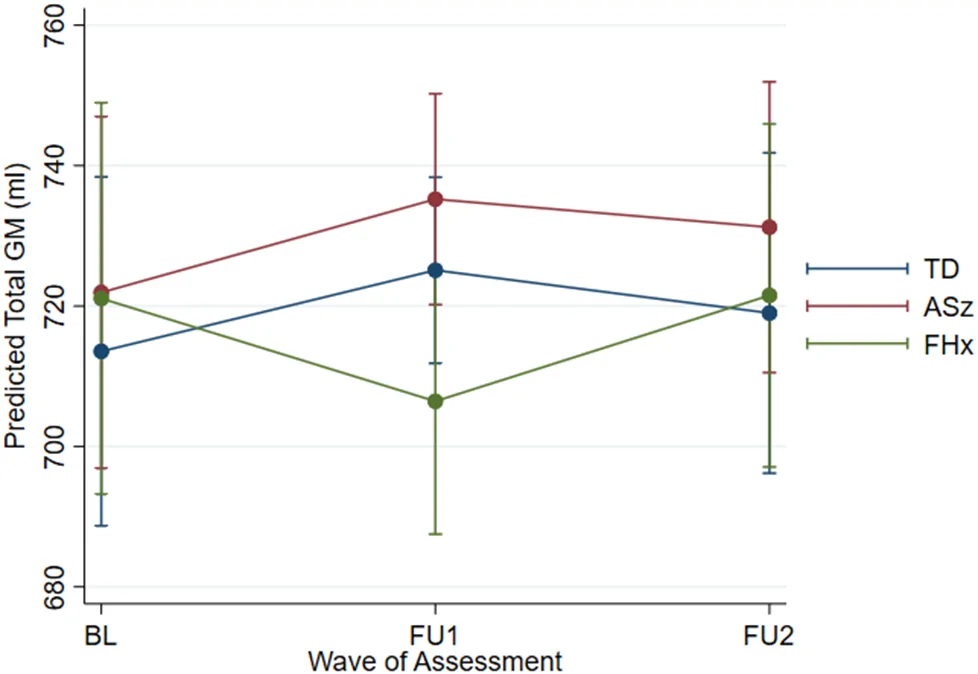
Margin plots of predicted total gray matter (GM) volume with 95% confidence intervals for the 3 groups showing changes over 3 waves of assessment, adjusted for age at baseline, days lapsing from baseline, sex, and intracranial volume. TD: typical development group; ASz: antecedent group; FHx: family history group; BL: baseline; FU1: follow-up 1; FU2: follow-up 2. Figure 2. long description.

We observed statistically significant group effects only in total WM volume (Figure 1), but not in total or any regional GM volumes (Figure 2). The follow-up pairwise analysis revealed that both ASz and FHx participants had higher WM volume compared to TD through all assessment waves (Figure 1).

Total WM and the ACC bilaterally showed a main effect of assessment wave. Here, regardless of group status, an increase in volume in total WM and both left and right ACC was observed at FU1 relative to BL (Figure 1, Supplementary Figures S1–S2), with the total WM and left ACC showing an increase at FU2 relative to BL. The effect in ACC did not survive correction. Other brain regions did not show a main effect of group or wave over the follow-up period.

### Group × time effect

There were no significant differences in volume change between assessment waves in any ROI in the ASz group compared to the TD group (**Table 2**, columns: ASz × FU1; and ASz × FU2). Conversely, there were several significant differences in time-related volume change in the FHx compared to the TD group (Table 2, columns: FHx × FU1; and FHx × FU2). For total WM, there was a continuous increase in both the TD and ASz groups from BL to FU2, while for FHx, total WM volume first decreased and then slightly increased. For volume of total GM, right hippocampus, and left and right middle temporal gyri, there was an increase in volume from BL to FU1 in the TD group, but a decrease in volume from BL to FU1 in the FHx group (Figure 2, Supplementary Figures S3–S5). Volume of the left insula remained almost the same from BL to FU2 in the TD group, but increased from BL to FU2 in the FHx group (Supplementary Figure S6). Finally, the right insula decreased in volume from BL to FU1 in the TD group, but increased in volume from BL to FU1 in the FHx group (Supplementary Figure S7). However, these effects in GM were no longer statistically significant after correction.

### Sensitivity and post hoc exploratory analysis

Sensitivity analyses were performed using the same models as in the main analyses, after reassigning the 5 individuals who met the criteria of both groups from the ASz to the FHx group. The results are shown in Supplementary Table S1. The result for total WM remained consistent with the main analysis; however, the difference between FHx and TD reduced in magnitude. The within-subject difference at both follow-up assessments in left and right ACC, and the impact of family history on left and right insula in the FHx group relative to TD, remained consistent with the main analyses. However, the group × wave differences in left and right MTG among the FHx group relative to TD were no longer significant.

We conducted a post hoc exploratory analysis to examine whether the persistence of antecedents was associated with differences in brain volumes within the ASz group. Sixteen individuals had persistent antecedents across both follow-up assessments (ASz+) and 16 did not (ASz−). Given the limited sample size, and to reduce measurement noise expected in smaller regions, we restricted this analysis to total GM and WM volumes rather than regional GM measures. Results were broadly consistent with the main analyses (Supplementary Table S2): no significant group differences were observed in total GM volume (Supplementary Figure S8), while total WM volume was higher in the ASz+ group compared with the ASz− group at follow-up (Supplementary Figure S9).

## Discussion

This study explored trajectories of brain structure development over approximately 4 years from ~11 years of age among 2 groups of at-risk children compared to their typically developing peers. Overall, the at-risk children exhibited larger WM volume. In terms of the developmental trajectory, the ASz group showed a similar pattern of brain development to the TD group in total WM and both total and regional GM volume (over the BL, FU1, and FU2 waves of assessment). In contrast, the FHx group showed altered trajectories in the right hippocampus, left and right insula, and left and right STG, albeit these effects attenuated following correction for multiple testing. The findings of this preliminary investigation provide initial insights, but they should be interpreted with caution given the modest sample size and limited statistical power.

Our findings of higher total WM volume align with our previous baseline VBM study,^28^ and with the Philadelphia Neurodevelopmental Cohort (PNC), which found greater WM volume in youths aged 8–22 years with psychotic spectrum symptoms.^41^ However, that difference was not significant when ICV was excluded. Additionally, the PNC study reported significantly lower GM volume regardless of ICV, which was not observed in our study. Notably, participants in this study were younger on average (~11 years old at BL) compared to those in the cross-sectional PNC study (~15 years old) where the difference in GM volume between youths with psychotic spectrum symptoms and controls became more pronounced with age. In the PNC study, when participants under 11 years were excluded, that difference became more prominent, suggesting that age at assessment significantly influences the observed disparities in brain volume.

Beyond morphometry differences, WM microstructure differences have been reported in adolescents with psychotic-like experiences.^42^ While the mechanisms underlying these differences are unclear, diffusion imaging studies suggest that inflammatory processes may be associated with increased extracellular water and thus play a role.^43^^,^^44^ As such, it is possible that WM volume changes (which we observed in this study) are related to inflammation; however, as our study does not include measures of diffusion metrics, free water imaging, or inflammatory markers, this explanation is speculative.

Regarding regional GM volumes, no main effects indicative of group differences were found (ASz vs. TD; and FHx vs. TD). However, we did observe time-related volume changes in the FHx group. Studies investigating regional brain structure in young people with psychotic-like experiences and genetic risk for psychosis have yielded varying results, including both increased and decreased regional GM volumes relative to their healthy peers.^45^^–^^48^ In our study, brain volume differences in the FHx group compared to TD children were most prominent at FU1 when the participants were around 13 years old, on average. Nonpsychotic siblings of patients with childhood-onset schizophrenia showed regional GM thickness deficits in childhood and early adolescence, but these abnormalities normalized by late adolescence (~17 years old).^49^^,^^50^ Together, these results suggest that the volumetric and trajectory differences observed among individuals with a family history of illness might vary with age, with adolescence representing a period of more marked deviance from “typical” development. However, the mechanisms underlying these changes are still unclear. Although the GM differences that we observed in the FHx group did not remain significant after correction for multiple testing, this should not be interpreted as evidence that no developmental or pathological alterations are present during this period. In addition, unmeasured factors such as IQ, socioeconomic status, and childhood adversity may contribute to the observed differences in brain structure. Larger studies are needed to replicate and further investigate these findings.

Sensitivity analyses that reassigned the 5 participants who met both ASz and FHx criteria from the ASz group to the FHx group revealed similar results as the main analysis for the effect of ASz. However, the effect of the FHx group on the trajectories of total GM and MTG and hippocampus was no longer significant. This finding may relate to the degree of relatedness within the sample, as 4 of the 5 participants had only 1 second-degree relative. Previous CHADS studies reported more pronounced neurocognitive impairments among individuals with stronger genetic relatedness.^51^^,^^52^ Unfortunately, our study was underpowered to examine differences in brain structure according to degree of relatedness.

Although both the ASz and FHx groups in our study were recruited from the general (nonhelp-seeking) population and may be considered at a very early at-risk state,^53^ detectable brain alterations were already present. The current analyses were not designed to model normative developmental trajectories in detail. Our aim was to examine whether brain features previously reported in association with schizophrenia could also be observed at an earlier stage. We therefore modelled assessment wave as a proxy for time since risk-status determination, particularly because ASz status was based on psychopathology measures that may vary over time. We acknowledge that this approach may reduce biological precision. Therefore, the findings should be interpreted with caution, particularly in distinguishing normative developmental variability from potential risk-related alterations. Future research adopting a developmental framework may further clarify how developmental and pathological trajectories interact across childhood and adolescence.

Prior CHADS work has identified abnormalities in a range of domains. For example, at-risk children show cognitive impairments, with ASz children displaying features closer to schizophrenia.^51^^,^^52^ Laurens et al.^29^ revealed ASz and FHx children had different developmental trajectories as indexed by event-related potential measures of auditory P3a amplitude. Notably, mismatch negativity abnormalities appeared only in ASz children with persistent symptoms. Our post hoc analysis incorporating persistence yielded similar results to the main total GM analysis, though with halved sample size. Fewer than 10 participants (aged 17–20 years) in our cohort met CHR criteria when reassessed,^54^ limiting our ability to evaluate predictive value. It is also important to note that not all individuals follow a prodromal pathway before developing schizophrenia.^55^ Thus, while larger prospective cohorts with longer follow-up will be required to address this question, the alterations identified here may still provide valuable insights into the underlying processes associated with psychosis risk.

Additional limitations of our study should be noted. First, our study had a small sample size of only 88 participants. When divided into 3 groups, the size of each group may have impeded our ability to identify subtle aberrations of brain structure, and precluded capacity to examine additional covariates such as cognitive functioning or IQ, childhood adversities, and socioeconomic status. These factors may operate as confounders, for example, lower IQ is associated with smaller GM and WM volumes.^56^ Although the evidence linking these covariates to brain structure is less consistent, they may contribute to the observed differences, and the findings might not be specific to psychosis risk. The sample size may also reduce the stability of interaction effects (ie, group × time). Although mixed effects models were employed to use all available data, these findings should be interpreted with caution, and replication in larger cohorts with longer follow-up is essential. Second, groups in our study were defined according to their status at baseline; however, persistent PLEs may be a more robust indicator of psychosis risk.^57^ For example, using larger samples from the CHADS cohort with standardized cognitive assessments available, Carpendale et al. reported differences in verbal learning,^51^ and persisting developmental deficits in IQ,^58^ among children with persistent ASz features. They also identified persisting developmental deficits in IQ among FHx children according to the combination of degree of family history (first- vs. second-degree relative) and presence of antecedents (none/1 vs. 2/3).^58^ However, we did not attempt equivalent analyses of ASz features within the FHx group in this small sample with imaging data available. Third, we applied the FreeSurfer recon default pipeline rather than a child-specific atlas for the study, even though the baseline scan was obtained between ages 9 and 12 years. Nonetheless, previous studies have shown that the default pipeline is appropriate for analyzing children,^59^ and has considerable reliability in longitudinal analysis of brain volume.^60^ Finally, while our study investigated the longitudinal brain structural changes in ASz children identified using a novel community screening approach, it remains unclear how these trajectories relate to progression to prodromal or full-threshold psychosis. Additional longitudinal studies are required to investigate brain development in the presence of particular risk factors of schizophrenia and the association with the outcome of these at-risk children.

In summary, our longitudinal MRI study of at-risk children revealed that both those presenting with a triad of antecedents of schizophrenia and those with a family history of schizophrenia exhibit altered global WM volumes compared to their typically developing peers. Furthermore, children with a family history of schizophrenia showed different GM volume trajectories at 2-year follow-up but with a potential tendency to normalize over time. Despite the small sample size, our findings underscore the importance of early screening in nonhelp-seeking populations. Importantly, there is a need to establish systematic follow-up assessments and tailored intervention strategies that can more accurately evaluate and stratify the risk of developing schizophrenia in these children, contributing to a more effective framework for early intervention. Further delineation of the relationship of phenotypic and genotypic risk factors with brain alterations is needed.

## Supporting information

10.1017/S1092852926101047.sm001 Si et al. supplementary material Si et al. supplementary material

## Data Availability

The data analyzed in this study is not publicly available but may be available for collaborative work from the corresponding author on reasonable request.

## Supplementary material

The supplementary material for this article can be found at http://doi.org/10.1017/S1092852926101047.

## Long descriptions

### Table 1. Long description

The table consists of five columns: Category, T D (n equals 35), A Sz (n equals 32), F H x (n equals 21), and p-value.

1. Number of participants with an M R I scan available, by assessment wave:

* B L: T D 21 (60 percent), A Sz 24 (75 percent), F H x 12 (57 percent).

* F U 1 (approximately 2 years): T D 32 (91 percent), A Sz 23 (72 percent), F H x 14 (67 percent).

* F U 2 (approximately 4 years): T D 30 (86 percent), A Sz 26 (81 percent), F H x 14 (67 percent).

2. Sex, female (percent):

* T D 20 (57 percent), A Sz 10 (31 percent), F H x 12 (57 percent), p-value 0.07.

3. Age (years), mean plus or minus S D:

* B L: T D 11.03 plus or minus 0.14, A Sz 11.18 plus or minus 0.24, F H x 11.56 plus or minus 0.27, p-value 0.15.

* F U 1 (approximately 2 years): T D 12.99 plus or minus 0.17, A Sz 12.81 plus or minus 0.25, F H x 13.52 plus or minus 0.20, p-value 0.07.

* F U 2 (approximately 4 years): T D 14.78 plus or minus 0.19, A Sz 14.50 plus or minus 0.22, F H x 15.03 plus or minus 0.22, p-value 0.23.

Abbreviations: T D, typically developing group; A Sz, antecedent group; F H x, family history group; B L, baseline; F U 1, follow-up 1; F U 2, follow-up 2.

Navigate back to Table 1..

### Table 2. Long description

The table is structured with 14 rows of brain regions and 17 columns of statistical data.

Header Structure:

- Regions of interest: The leftmost column.

- R super 2: Conditional and Marginal values.

- Group effect: Estimated between-group differences over 4 years (A S z versus T D, and F H x versus T D).

- Wave effect: Estimated change over assessment waves (F U 1 versus B L, and F U 2 versus B L).

- Group by wave interaction: Estimated differences in change between waves for A S z and F H x groups.

Key Data Points:

- Total G M: Conditional R super 2 is 0.89. A significant negative interaction for F H x by F U 1 is noted at -26.200.

- Total W M: Conditional R super 2 is 0.99. Significant positive group effects for A S z (29.452) and F H x (36.494) versus T D. Significant wave effects for F U 1 (15.262) and F U 2 (23.809) versus B L. Significant negative interactions for F H x at F U 1 (-11.813) and F U 2 (-14.549).

- Left and Right Hippocampus: Marginal R super 2 values are 0.45 and 0.43 respectively. Most interaction terms are listed as N A.

- Left and Right Insula: Significant positive interactions for F H x by F U 2 in the Left Insula (0.246) and F H x by F U 1 in the Right Insula (0.364).

- Left and Right A C C: Significant wave effects for F U 1 (0.273 and 0.176) and F U 2 (0.408 for Left A C C).

- Left and Right M T G: Significant negative interactions for F H x by F U 1 (-1.062 and -1.124).

Abbreviations: T D (typically developing), A S z (antecedent group), F H x (family history), B L (baseline), F U (follow-up), G M (gray matter), W M (white matter), A C C (anterior cingulate), M T G (middle temporal gyrus), S T G (superior temporal gyrus).

Navigate back to Table 2..

### Figure 1. Long description

The x-axis is labeled Wave of Assessment with three points: B L, F U 1, and F U 2. The y-axis is labeled Predicted Total W M in milliliters, ranging from 400 to 480. Three colored lines with error bars represent the groups.

* The T D group, represented by a blue line, starts at the lowest point at B L with approximately 415 milliliters and shows a steady linear increase to roughly 430 at F U 1 and 438 at F U 2.

* The A S z group, represented by a red line, starts at approximately 444 milliliters at B L, rises sharply to 458 at F U 1, and continues to 465 at F U 2.

* The F H x group, represented by a green line, starts at approximately 451 milliliters at B L, showing a more gradual linear increase to 454 at F U 1 and 460 at F U 2.

Vertical error bars representing 95 percent confidence intervals are present at each data point for all three groups. A legend on the right identifies the blue line as T D, the red line as A S z, and the green line as F H x.

Navigate back to Figure 1..

### Figure 2. Long description

The x-axis is labeled Wave of Assessment with three discrete points: B L, F U 1, and F U 2. The y-axis is labeled Predicted Total G M in milliliters, ranging from 680 to 760 in increments of 20.

* The T D group, represented by a blue line with circular markers, starts at approximately 714 at B L, increases to 725 at F U 1, and slightly decreases to 719 at F U 2.

* The A S z group, represented by a red line with circular markers, starts at approximately 722 at B L, increases to 735 at F U 1, and decreases to 731 at F U 2.

* The F H x group, represented by a green line with circular markers, starts at approximately 721 at B L, decreases to 707 at F U 1, and increases back to 721 at F U 2.

Each data point includes vertical error bars representing 95 percent confidence intervals. A legend on the right identifies the three groups: T D in blue, A S z in red, and F H x in green.

Navigate back to Figure 2..
