# Supplementary material for: Trajectories of gray and white matter volume in children at elevated risk for schizophrenia
Source: CNS Spectr. 2026 Jun 9;31(1):e23. doi: 10.1017/S1092852926101047 (PMC13732592; doi:10.1017/S1092852926101047)
Supplement: Supplementary file 1 [file S1092852926101047sup001.docx]

# Supplementary Methods

**Definition of the Triad of putative antecedents^1^:**

(1) Caregiver-reports of delays or abnormalities in speech or motor development: The caregiver completed three items concerning the age at which the child achieved key motor (walking unassisted) and speech (using single words; using two- and three-word phrases) milestones, five questions about personal or professional concern regarding delays or abnormalities in motor or speech development (e.g., standing, walking, speaking), and one item indicating whether the child presently experienced difficulty with co-ordination or unsteadiness. A speech problem was coded as present whenever either a speech delay or a parental or professional expression of concern about speech development during the child's first three years of life was reported. A motor problem was coded as present when either a motor delay or a parental or professional expression of concern regarding attainment of motor milestones during the child's first three years of life or a current difficulty with motor co-ordination or unsteadiness was reported.
(2) Caregiver-reports of externalising and peer problems, and child-reports of emotional symptoms: children and caregivers completed the self- and parent-report versions respectively of the Strengths and Difficulties Questionnaire^2^ to assess four domains of child psychopathology (Emotional Symptoms, Conduct Problems, Hyperactivity-Inattention, and Peer Problems) as well as personal strengths (Prosocial Behaviour).
(3) Child-reports of PLEs: PLEs were assessed using the 9 items of the Psychotic-Like Experiences Questionnaire for Children (PLEQ-C)^1,3,4^: (a) “Some people believe that their thoughts can be read. Have other people ever read your thoughts?”, (b) “Have you ever believed that you were being sent special messages through the television”, (c) “Have you ever thought that you were being followed or spied upon?”, (d) “Have you ever heard voices that other people could not hear?”, (e) “Have you ever felt as though your body had been changed in some way that you could not understand?”, (f) “Have you ever felt that you were under the control of some special power?”, (g) “Have you ever known what another person was thinking even though that person wasn't speaking?”, (h) “Do you have any special powers that other people don't have?”, and (i) “Have you ever seen something or someone that other people could not see?”.

**Inclusion of FHx group:** Family history of schizophrenia or schizoaffective disorder was obtained from caregiver questionnaires or from patients' medical records within the South London and Maudsley (SLaM) National Health Service (NHS) Foundation Trust, and confirmed using the Family Interview for Genetic Studies (FIGS)^5^. Children with family history of bipolar disorder (without family history of schizophrenia/schizoaffective disorder) were initially sought as a separate group of potential interest, but with insufficient recruits, these data were not analysed ^5^.

# Figure S1

**Figure S1.** Margins plot of predicted left anterior cingulate cortex (ACC) volume changes over three waves of assessment in the three risk groups adjusted for age at baseline, days from baseline, sex, and total intracranial volume. TD: Typical development group; ASz: Antecedent group; FHx: Family history group; BL: baseline; FU1: Follow up 1; FU2: Follow up 2.

# Figure S2

**Figure S2**. Margins plot of predicted right anterior cingulate cortex (ACC) volume changes over three waves of assessment in the three risk groups, adjusted for age at baseline, days from baseline, sex, and total intracranial volume. TD: Typical development group; ASz: Antecedent group; FHx: Family history group; BL: baseline; FU1: Follow up 1; FU2: Follow up 2.

# Figure S3

**Figure S3.** Margins plot of predicted right hippocampus volume changes over three waves of assessment in the three risk groups adjusted for age at baseline, days from baseline, sex, and total intracranial volume. TD: Typical development group; ASz: Antecedent group; FHx: Family history group; BL: baseline; FU1: Follow up 1; FU2: Follow up 2.

# Figure S4

**Figure S4.** Margins plot of predicted left middle temporal gyrus (MTG) volume changes over three waves of assessment in the three risk groups, adjusted for age at baseline, days from baseline, sex, and total intracranial volume. TD: Typical development group; ASz: Antecedent group; FHx: Family history group; BL: baseline; FU1: Follow up 1; FU2: Follow up 2.

# Figure S5

**Figure S5.** Margins plot of predicted right middle temporal gyrus (MTG) volume changes over three waves of assessment in the three risk groups adjusted for age at baseline, days from baseline, sex, and total intracranial volume. TD: Typical development group; ASz: Antecedent group; FHx: Family history group; BL: baseline; FU1: Follow up 1; FU2: Follow up 2.

# Figure S6

**Figure S6.** Margins plot of predicted left insula volume changes over three waves of assessment in the three risk groups, adjusted for age at baseline, days from baseline, sex, and total intracranial volume. TD: Typical development group; ASz: Antecedent group; FHx: Family history group; BL: baseline; FU1: Follow up 1; FU2: Follow up 2.

# Figure S7

**Figure S7.** Margins plot of predicted right insula volume changes over three waves of assessment in the three risk groups, adjusted for age at baseline, days from baseline, sex, and total intracranial volume. TD: Typical development group; ASz: Antecedent group; FHx: Family history group; BL: baseline; FU1: Follow up 1; FU2: Follow up 2.

Table S1 Parameter estimates of the effect of risk group on grey matter volume over three waves of assessment in sensitivity analyses that reassigned the five participants who met both ASz and FHx criteria from the ASz to the FHx group.

| **Regions of interest** | **Estimated between-group differences over 4 years** **(Group effect)** | | | | **Estimated change at every follow-up timepoint**  **(Assessment wave effect)** | | | | **Estimated between-group differences in change between respective assessment waves**  **(Group x Wave of Assessment)** | | | | | | | |
| --- | --- | --- | --- | --- | --- | --- | --- | --- | --- | --- | --- | --- | --- | --- | --- | --- |
|  | **TD vs ASz** | | **TD vs FHx** | | **FU1 vs BL** | | **FU2 vs BL** | | **ASz x FU1** | | **ASz x FU2** | | **FHx x FU1** | | **FHx x FU2** | |
|  | ***b*** | ***95CI*** | ***b*** | ***95CI*** | ***b*** | ***95CI*** | ***b*** | ***95CI*** | ***b*** | ***95CI*** | ***b*** | ***95CI*** | ***b*** | ***95CI*** | ***b*** | ***95CI*** |
| **Total GM** | 8.564 | -14.044 to 31.172 | 5.145 | -18.083 to 28.373 | 12.906 | -12.012 to 37.825 | 8.036 | -33.168 to 49.240 | -2.064 | -24.229 to 20.101 | 3.436 | -18.820 to 25.692 | 13.163 | -35.695 to 9.368 | -1.186 | -23.906 to 21.535 |
| **Total WM** | **31.594**** | 16.380 to 46.808 | **31.399**** | 16.030 to 46.768 | **14.938**** | 5.882 to 23.994 | **23.195**** | 7.645 to 38.746 | -0.635 | -7.402 to 6.132 | -3.364 | -10.196 to 3.469 | **-7.624*** | -14.542 to -0.707 | **-9.693**** | -16.630 to -2.756 |
| **Left hippocampus** | -0.024 | -0.205 to 0.156 | -0.085 | -0.266 to 0.097 | -0.064 | -0.198 to 0.070 | -0.169 | -0.408 to 0.069 | NA | NA | NA | NA | NA | NA | NA | NA |
| **Right hippocampus** | 0.014 | -0.204 to 0.232 | -0.022 | -0.242 to 0.199 | 0.087 | -0.074 to 0.247 | 0.073 | -0.201 to 0.346 | -0.027 | -0.150 to 0.096 | 0.006 | -0.118 to 0.130 | -0.113 | -0.239 to 0.012 | -0.023 | -0.149 to 0.103 |
| **Left fusiform** | 0.060 | -0.584 to 0.704 | -0.007 | -0.654 to 0.640 | 0.211 | -0.193 to 0.616 | 0.106 | -0.616 to 0.828 | NA | NA | NA | NA | NA | NA | NA | NA |
| **Right fusiform** | 0.290 | -0.277 to 0.857 | 0.296 | -0.274 to 0.866 | 0.045 | -0.384 to 0.475 | -0.288 | -1.052 to 0.476 | NA | NA | NA | NA | NA | NA | NA | NA |
| **Left insula** | 0.113 | -0.216 to 0.442 | 0.152 | -0.181 to 0.486 | 0.044 | -0.213 to 0.301 | -0.037 | -0.475 to 0.401 | 0.029 | -0.171 to 0.228 | 0.131 | -0.070 to 0.332 | 0.177 | -0.027 to 0.380 | **0.213*** | 0.009 to 0.418 |
| **Right insula** | 0.010 | -0.343 to 0.362 | 0.151 | -0.208 to 0.510 | -0.258 | -0.560 to 0.044 | -0.465 | -0.979 to 0.048 | 0.058 | -0.181 to 0.298 | 0.102 | -0.140 to 0.344 | **0.320*** | 0.075 to 0.564 | 0.179 | -0.067 to 0.425 |
| **Left ACC** | -0.019 | -0.468 to 0.431 | 0.272 | -0.180 to 0.724 | **0.274*** | 0.048 to 0.499 | **0.410*** | 0.006 to 0.813 | NA | NA | NA | NA | NA | NA | NA | NA |
| **Right ACC** | 0.115 | -0.282 to 0.512 | -0.024 | -0.422 to 0.375 | **0.174*** | 0.006 to 0.343 | 0.190 | -0.112 to 0.493 | NA | NA | NA | NA | NA | NA | NA | NA |
| **Left MTG** | 0.090 | -0.857 to 1.036 | 0.125 | -0.844 to 1.094 | 0.449 | -0.525 to 1.422 | 0.024 | -1.604 to 1.653 | 0.203 | -0.624 to 1.031 | 0.290 | -0.543 to 1.122 | -0.566 | -1.409 to 0.277 | -0.206 | -1.055 to 0.642 |
| **Right MTG** | 0.626 | -0.460 to 1.713 | 0.123 | -0.987 to 1.233 | 0.758 | -0.314 to 1.830 | -0.001 | -1.803 to 1.801 | -0.341 | -1.235 to 0.552 | -0.179 | -1.078 to 0.720 | -0.523 | -1.434 to 0.387 | 0.069 | -0.847 to 0.985 |
| **Left STG** | 0.345 | -0.445 to 1.136 | 0.601 | -0.194 to 1.396 | 0.378 | -0.168 to 0.923 | 0.383 | -0.590 to 1.356 | NA | NA | NA | NA | NA | NA | NA | NA |
| **Right STG** | 0.250 | -0.499 to 0.999 | 0.643 | -0.110 to 1.395 | 0.090 | -0.418 to 0.598 | -0.082 | -0.987 to 0.824 | NA | NA | NA | NA | NA | NA | NA | NA |

**p*<0.05; ***p*<0.01; TD: Typically developing group; ASz: Antecedent group; FHx: Family history group; NA: Interaction between group and time was not significant and dropped from the model; GM: Total grey matter volume; WM: Total white matter volume; ACC: Anterior cingulate; MTG: Middle temporal gyrus; STG: Superior temporal gyrus; BL: baseline; FU1: Follow up 1; FU2: Follow up 2.

# Post-hoc exploratory analysis

# Table S2 Parameter estimates of the effect of the persistence of antecedents on grey matter volume over three waves of assessment in post-hoc analyses.

| **Regions of interest** | **Estimated between-group differences over 4 years** **(Group effect)** | | | | **Estimated change at every follow-up timepoint**  **(Assessment wave effect)** | | | | **Estimated between-group differences in change between respective assessment waves**  **(Group x Wave of Assessment)** | | | | | | | |
| --- | --- | --- | --- | --- | --- | --- | --- | --- | --- | --- | --- | --- | --- | --- | --- | --- |
|  | **TD vs ASz+** | | **TD vs ASz-** | | **FU1 vs BL** | | **FU2 vs BL** | | **ASz+ x FU1** | | **ASz+ x FU2** | | **ASz- x FU1** | | **ASz- x FU2** | |
|  | ***b*** | ***95CI*** | ***b*** | ***95CI*** | ***b*** | ***95CI*** | ***b*** | ***95CI*** | ***b*** | ***95CI*** | ***b*** | ***95CI*** | ***b*** | ***95CI*** | ***b*** | ***95CI*** |
| **Total GM** | 14.255 | -6.332 to 34.842 | 7.056 | -13.982 to 28.094 | 14.698 | -2.751 to 32.146 | 10.716 | -20.679 to 42.113 | NA | NA | NA | NA | NA | NA | NA | NA |
| **Total WM** | **34.714**** | 17.829 to 51.600 | **21.124*** | 3.839 to 38.409 | **15.243**** | 6.179 to 24.308 | **23.191**** | 6.729 to 39.654 | NA | NA | NA | NA | NA | NA | NA | NA |

**p*<0.05; ***p*<0.01; TD: Typically developing group; ASz+: Persistent antecedents group; ASz-: Non-persistent antecedents group; NA: Interaction between group and time was not significant and dropped from the model; GM: Total grey matter volume; WM: Total white matter volume; BL: baseline; FU1: Follow up 1; FU2: Follow up 2.

# Figure S8

**Figure S8.** Margins plot of predicted total grey matter volume changes over three waves of assessment in the three risk groups, adjusted for age at baseline, days from baseline, sex, and total intracranial volume. TD: Typical development group; ASz+: Persistent antecedents group; ASz-: Non-persistent antecedents group; BL: baseline; FU1: Follow up 1; FU2: Follow up 2.

# Figure S9

**Figure S9.** Margins plot of predicted total white matter volume changes over three waves of assessment in the three risk groups, adjusted for age at baseline, days from baseline, sex, and total intracranial volume. TD: Typical development group; ASz+: Persistent antecedents group; ASz-: Non-persistent antecedents group; BL: baseline; FU1: Follow up 1; FU2: Follow up 2.

# References

1.	Laurens KR, Hodgins S, Maughan B, Murray RM, Rutter ML, Taylor EA. Community screening for psychotic-like experiences and other putative antecedents of schizophrenia in children aged 9–12 years. *Schizophrenia Research*. 2007/02/01/ 2007;90(1):130-146. doi:<https://doi.org/10.1016/j.schres.2006.11.006>

2.	Goodman R, Meltzer H, Bailey V. The Strengths and Difficulties Questionnaire: A pilot study on the validity of the self-report version. *European child & adolescent psychiatry*. 1998;7(3):125-130.

3.	Gutteridge TP, Lang CP, Turner AM, Jacobs BW, Laurens KR. Criterion validity of the Psychotic-Like Experiences Questionnaire for Children (PLEQ-C). *Schizophr Res*. Jun 2020;220:78-84. doi:10.1016/j.schres.2020.03.067

4.	Laurens KR, Hobbs MJ, Sunderland M, Green MJ, Mould GL. Psychotic-like experiences in a community sample of 8000 children aged 9 to 11 years: an item response theory analysis. *Psychological Medicine*. 2012;42(7):1495-1506. doi:10.1017/S0033291711002108

5.	Laurens KR, Cullen AE. Toward earlier identification and preventative intervention in schizophrenia: evidence from the London Child Health and Development Study. *Social Psychiatry and Psychiatric Epidemiology*. 2016/04/01 2016;51(4):475-491. doi:10.1007/s00127-015-1151-x
